# Runx1t1 promotes the neuronal differentiation in rat hippocampus

**DOI:** 10.1186/s13287-020-01667-x

**Published:** 2020-04-22

**Authors:** Linqing Zou, Haoming Li, Xiao Han, Jianbing Qin, Guoqi Song

**Affiliations:** 1 Department of Human Anatomy, Jiangsu Key Laboratory of Neuroregeneration, Nantong University, Nantong, 226001 Jiangsu China; 2 Department of Hematology, Affiliated Hospital of Nantong University, Nantong, 226001 Jiangsu China; 3 Department of Medical Oncology, Sidney Kimmel Cancer Center, Thomas Jefferson University, Philadelphia, PA 19107 USA; 4 Department of Neurochemistry, Inge Grundke-Iqbal Research Floor, New York State Institute for Basic Research in Developmental Disabilities, Staten Island, NY USA

**Keywords:** Runx1t1, Hippocampus, Neural stem cells, Neuron, Differentiation, Neurogenesis

## Abstract

**Background:**

Runt-related transcription factor 1 translocated to 1 (Runx1t1) is one of the members of the myeloid translocation gene family. Our previous work showed that Runx1t1 induced the neuronal differentiation of radial glia cells in vitro.

**Methods:**

To better uncover the role of Runx1t1 in hippocampal neurogenesis, in this study, we further explore its localization and function during the hippocampal neurogenesis.

**Results:**

Our results showed that insufficient expression of Runx1t1 reduced the neuronal differentiation, and overexpression of Runx1t1 promoted the neuronal differentiation in vitro. We also found that Runx1t1 localized in neurons but not astrocytes both in vivo and in vitro. Furthermore, we found that Runx1t1 overexpression elevated the number of newborn neurons in the hippocampal dentate gyrus.

**Conclusions:**

Taken together, our results further proved that Runx1t1 could be worked as a regulator in the process of hippocampal neurogenesis.

## Introduction

Neurogenesis is a process by which newborn neurons are generated from neural stem cells (NSCs) or neural progenitor cells (NPCs); NSCs were considered to be a potential source of cells for cell replacement therapy during brain damage repair [1–3]. However, the usefulness of the strategies has been obstructed, especially, by limited neuronal differentiation of NSCs [1, 4, 5]. Thus, the identification of the factors and mechanisms underlying the neuronal differentiation of NSCs in guiding the production of NSCs for clinical needs is imperative.

Neurogenesis in the dentate gyrus (DG) of hippocampus can persist throughout the whole life [5–8], and it is also a multiple-step process and is regulated by several intrinsic and extrinsic factors [9, 10]. During the neurogenesis, a cascade of gene expression activated by proneural basic helix-loop-helix (bHLH) proteins plays an essential and conserved role in promoting neuronal differentiation [11, 12], such as myeloid translocation genes (MTGs). MTG proteins have been reported to be sequentially expressed during neuronal differentiation and may promote the transition from precursor to neuron and induce the expression of neuronal genes in the differentiated cells [13]. Some reports using biochemical and molecular analyses suggest that MTG family members act as downstream of proneural proteins to regulate stem cell proliferation and promote neuronal differentiation [14–16].

Runt-related transcription factor 1 translocated to 1 (Runx1t1) is one of the members of the MTG family. It was reported that Runx1t1 was involved in the proliferation and differentiation of hematopoietic stem cells [17, 18]. However, the exact function of Runx1t1 in neural development is largely unexplored. In present work, the expression and distribution of Runx1t1 were detected during the differentiation of NSCs from rat hippocampus in vitro and in hippocampal DG in vivo. We found that Runx1t1 was strongly expressed in NSCs and weakly detectable in neurons in vitro and in vivo. Moreover, upregulation of Runx1t1 promoted hippocampal neuronal differentiation both in vitro and in vivo. Taken together, our results suggested that Runx1t1 was closely related to the neural differentiation in hippocampus.

## Materials and methods

### NSC culture and identification

NSCs were acquired as previously described [8, 19, 20]. Animal experiments were conducted according to the protocols approved by the United States National Institutes of Health Guide for the Care and Use of Laboratory Animals. All efforts were made to minimize the number and suffering of animals used in this study. Briefly, embryos were harvested from pregnant rats on embryonic day 16 (E16). The embryonic hippocampal were immediately dissected, isolated, and triturated into single-cell suspensions. After centrifugation, the cells were resuspended and maintained at a density of 1 × 10^5^ in 10 ml Dulbecco’s modified Eagle medium (DMEM)/F12 containing 2% B27, 20 ng/ml epidermal growth factor (EGF), and fibroblast growth factor (FGF-2; Sigma), which is a neurosphere expansion medium. Seven days later, the primary neurospheres were passaged by the dissociation of bulk neurospheres using Accutase (Sigma). After three passages (P3), the neurospheres were processed for immunocytochemistry to identify their stem/progenitor properties. Besides, 5 nM 5-bromo-2-deoxy-uridine (BrdU; Roche, Mannheim, Germany) was added to the medium for 24 h to label proliferated cells in vitro. The P3 neurospheres were triturated into single-cell suspensions, at a density of 1.5 × 10^4^ cells/mL in 24-well plates containing the differentiation medium (DMEM/F12 medium with 2% B27 and 2% fetal bovine serum (FBS; GIBCO)) for other experiments.

### Lentiviral vector construction and infection

The RNA interference (RNAi) lentiviral vector (LV3-Runx1t1-RNAi) was constructed using GV118 lentiviral expression system (GeneChem Co. Ltd., Shanghai, China) as described previously [21]. Briefly, a vector-based RNAi approach was used to produce intracellular short hairpin double-stranded RNA from a DNA template under the control of the pCMV promoter. The oligonucleotide sequence of shRNA is 5′-TAAGCAAGCGACCATGCACTATCTCGAGATAGTGCATGGTCGCTTGCTTTTTTTTC-3′.

The underlined letters denote the hairpin loop. The negative control (NC) sequence was 5′-CCGGTTCTCCGAACGTGTCACGTTTCAAGAGAACGTGACACGTTCGGAGAATTTTTG-3′. GV287 lentiviral expression system (GeneChem) was used to acquire the Runx1t1 overexpressing lentivirus LV4-Runx1t1 (2 × 10^8^ TU/mL) and the negative control lentivirus LV4-NC (1 × 10^9^ TU/mL) as previously described [21].

For the infection experiments, cells were divided into six groups. For siRNA interference, single cells were cultured in the differentiation medium containing 30 μL of 1 × 10^8^ TU/mL LV3-Runx1t1-RNAi or 10 μL of 3 × 10^8^ TU/mL LV3-NC with 8 μg/mL polybrene (GeneChem). Following incubation for 12 h, the culture medium was replaced with medium without lentivirus. For Runx1t1 overexpression, the cells were cultured in differentiation medium containing 20 μL of 2 × 10^8^ TU/mL LV4-Runx1t1 or 4 μL of 1 × 10^9^ TU/mL LV4-NC with 8 μg/mL polybrene for 24 h, and then, the medium was replaced by fresh medium. After 3 days, Runx1t1 protein and RNA expression in the NSCs was analyzed using real-time PCR and Western blot.

### Stereotactic injections

Eighteen adult Sprague-Dawley (SD) rats weighing 220–250 g were purchased from the Experimental Animal Center of Nantong University. The animals were maintained in a controlled temperature environment (23 ± 2 °C) on a 12 h to 12 h light to dark cycle in an approved facility with free access to food and water. After transient anesthesia with chlorpent (2 ml/kg body weight, i.p.), adult SD rats were transferred to the stereotaxic apparatus, LV4-Runx1t1 were injected into unilateral DG area, the negative control lentivirus were injected opposite. According to the atlas of Paxinos and Watson [22], the injection site was 1.8 mm lateral to midline, 3.3 mm posterior to bregma, and 4.4 mm inferior to the upper surface of the skull bone. Each side received 10 μl lentivirus. After suturing the skin and applying penicillin (100,000 units/kg, i.p.), the animals were allowed to recover. On day 3 after injection, real-time PCR and Western blot (6 rats respectively) were used to detect Runx1t1 RNA and protein expression. Fourteen days later, another 6 rats were perfused transcardially with 0.9% NaCl followed by chilled 4% paraformaldehyde (PFA) in 0.1 M phosphate-buffered saline (PBS, pH 7.4). Coronal frozen sections were sliced frontally at 30-μm thickness through the hippocampus; six sections encompassing the hippocampus were collected randomly from each animal for immunocytochemistry.

### RNA extraction and real-time PCR

Total RNA was isolated using a UNIQ-10 Spin Column RNA Purification Kit (Sangon, Shanghai, China). First-strand cDNA was synthesized using RevertAid™ First-Strand cDNA Synthesis Kit (Fermentas, Burlington, Canada). The first-strand cDNA was subsequently processed with the Corbett RG-6000 PCR system (Qiagen, Dusseldorf, German) using FastStart Universal SYBR Green Master Mix (Roche, Basel, Switzerland). The reactions were optimized by varying the annealing temperatures from 48 to 55 °C. The sense and antisense primers were synthesized as follows: GAPDH, 5′-GCAAGTTCAACGGCACAG-3′, 5′-GCCAGTAGACTCCACGACAT-3′; and Runx1t1, 5′-CCATTGCCCACCACTA-3′, 5′-CCACTCTTCTGCCCATT-3′.

### Western blot assay

Western blot analysis was performed as described previously [18]. Equivalent amounts of total protein from cells or hippocampal tissue were separated by SDS-PAGE and transferred to polyvinylidene fluoride (PVDF) membranes using Bio-Rad Semi-Dry Transfer Cell (Bio-Rad, CA, USA). The membranes were incubated with primary antibody rabbit anti-Runx1t1 (1:300; Abcam, Cambridge, UK) and mouse anti-β-actin (1:2000; Beyotime, Jiangsu, China), followed by the corresponding HRP-conjugated secondary antibodies (1:2000). The immunoreactive bands were scanned on a ChemiDoc XRS system (Bio-Rad), and the optical density was measured. The relative expression of the Runx1t1 protein in the different groups was determined semi-quantitatively using Quantity One software (Bio-Rad).

### Immunofluorescence staining analyses

Cells were fixed in 4% PFA, and brain sections were incubated with primary antibodies at 4 °C for 48 h, followed by overnight incubation with secondary antibodies conjugated to fluorescein 488 and 594 at 4 °C. The primary antibodies used were as follows: mouse anti-nestin (1:100), rat anti-BrdU (1:200), rat anti-Runx1t1 (1:300), guinea-pig anti-DCX (1:1000), mouse anti-MAP-2 (1:200), mouse anti Tuj1 (1:400), mouse anti NeuN (1:500), and rabbit anti-glial fibrillary acidic protein (GFAP; 1:1000). All the primary antibodies were purchased from Millipore (Billerica, MA, USA) and Abcam. The cell nuclei were counterstained with Hoechst (Sigma). After double- or triple-labeled immunofluorescence staining for cellular markers and enhanced green fluorescent protein (EGFP), the cells and sections were observed using an Olympus laser confocal microscope (Fv10i; Olympus, Tokyo, Japan). Positively stained cells were counted in five randomly selected microscopic visual fields/well.

### Statistical analysis

Data from the experiments were subjected to one-way analysis of variance (ANOVA) or Student’s *t* test using the SPSS 11.5 statistics software. All data were expressed as the mean ± SEM, and all experimental results were obtained from a minimum of three independent experiments.

## Results

### Decreased expression of Runx1t1 during the neuronal differentiation of NSCs

**Fig. 1:**
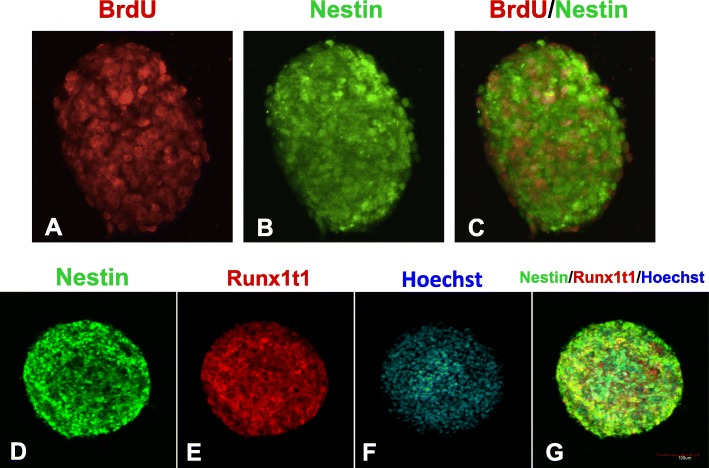
Hippocampal newly formed neurospheres acquired from neonatal rat hippocampus co-expressed with BrdU and nestin (**a**–**c**), and they can also be labeled by Runx1t1 (**d**–**g**) in vitro

**Fig. 2:**
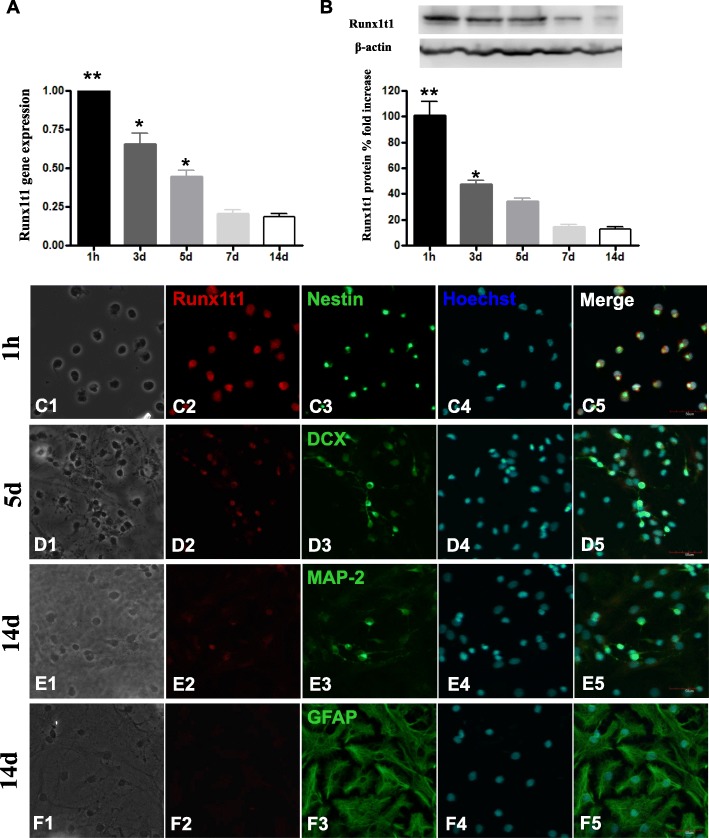
Runx1t1 gene expression level was the highest in the cells at 1 h after differentiation culture, started to decline after 3 and 5 days of differentiation, and maintained the low level at a later stage of differentiation (**A**). A similar expression pattern was observed for the Runx1t1 protein level (**B**). At 1 h after differentiation, approximately all cells strongly expressed Runx1t1 and were co-labeled by nestin (**C1**–**C5**). On day 5, the number of Runx1t1 immunopositive cells decreased significantly. Only DCX-positive cells co-expressed Runx1t1 (**D1**–**D5**). On day 14, Runx1t1 expression was still detected in the MAP 2-positive cells (**e**) but was undetectable in the GFAP-positive cells (**F1**–**F5**). Scale bars = 50 μm

After being cultured in NSC expansion medium, the neurospheres co-expressed BrdU with nestin (Fig. 1a–c) and was labeled with Runx1t1 (Fig. 1d, e). To assess the distribution of Runx1t1 during the differentiation of NSCs in vitro, neurospheres were dissociated into single cells and transferred into the basal differentiation medium (DMEM/F12 medium supplemented with 2% FBS). The expression of Runx1t1 gene and protein were detected at different time points. The results showed that the Runx1t1 gene expression level was reduced of neuronal differentiation at 3 days and 5 days and was maintained at a low level in the later stages of differentiation (Fig. 2A). A similar expression pattern was observed for the Runx1t1 protein (Fig. 2B).

To determine the localization of Runx1t1 expression during differentiation, we used DCX, MAP 2, and GFAP to co-label with Runx1t1. After treatment of differentiation culture medium for 1 h, most of the cells strongly expressed Runx1t1 and co-labeled with nestin (Fig. 2C1–C5). Five days later, the number of Runx1t1-positive cells decreased. Only DCX-positive cells co-expressed Runx1t1 (Fig. 2D1–D5). At 14 days after the cells differentiated, Runx1t1 was still detected in the MAP-2-positive cells (Fig. 2E1–E5), but Runx1t1-positive cells were undetectable in the GFAP-positive cells (Fig. 2F1–F5). Together, these results showed that Runx1t1 was strongly expressed in NSCs. But during the neural differentiation, the expression of Runx1t1 declined gradually and was detectable in neurons but not astrocytes. Thus, we hypothesized that Runx1t1 was involved in the neural differentiation of hippocampal NSCs.

### Runx1t1 regulates the neuronal differentiation in NSCs in vitro

**Fig. 3:**
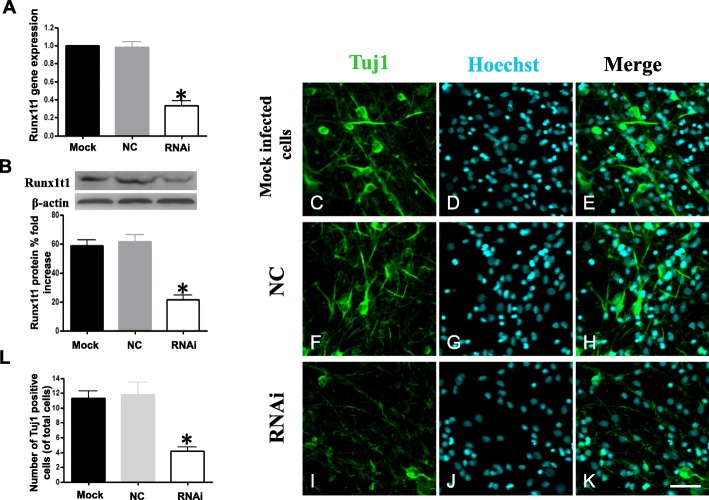
Knockdown of Runx1t1 decreased neuronal differentiation of hippocampal NSCs. The gene and protein expression of Runx1t1 in the RNAi group was significantly lower than the mock-infected and NC groups (**a**, **b**). Compared to the mock and NC groups, less Tuj1-positive cells were detected after treatment with LV3-Runx1t1-RNAi, and the length of processes of Tuj1-positive cells became shorter, lesser, and even less evident (**c**–**k**). Scale bar = 50 μm. The numbers of Tuj1-positive cells showed a significant difference between the mock-infected and LV3-NC groups with an LV3-Runx1t1-RNAi group (**l**). *P* < 0.05. NC, negative control; RNAi, RNA interference

**Fig. 4:**
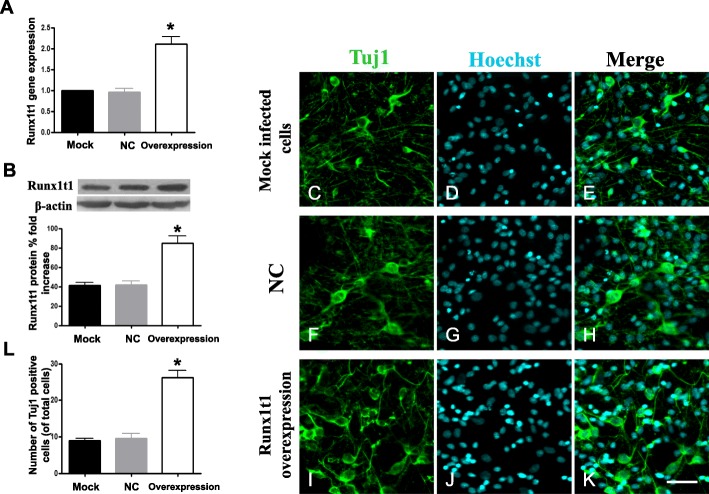
Runx1t1 gene expression level in the Runx1t1 overexpression group was higher than that in the mock-infected and NC groups (**a**). The level of Runx1t1 protein in the Runx1t1 overexpression group was also significantly greater than that in the mock-infected and LV4-NC groups (**b**). The difference between the Runx1t1 overexpression group and the other groups was statistically significant. Thus, the NSCs from the hippocampus infected with LV4-Runx1t1 showed effectively upregulated Runx1t1 protein and gene expression. Compared to the mock-infected and LV4-NC groups, a large number of Tuj1-positive cells were detected in LV4-Runx1t1, and the length of processes of Tuj1-positive cells increased (**a**–**c**). Scale bar = 50 μm. The numbers of Tuj1-positive cells showed a significant difference between mock-infected and LV4-NC groups with an LV4-Runx1t1 group (**l**). *P* < 0.05

Then, we knocked down Runx1t1 in NSCs with Runx1t1 RNAi using lentiviral vectors to determine the roles of Runx1t1 during NSCs differentiation. Three days after the Runx1t1 knockdown, real-time PCR showed that Runx1t1-RNAi caused Runx1t1 to be to be significantly knocked down (Fig. 3a). Among the blank, LV3-NC, and LV3-Runx1t1-RNAi groups, the expression level of Runx1t1 gene in the LV3-Runx1t1-RNAi group was markedly lowest. The difference between the LV3-Runx1t1-RNAi and mock-infected cells/LV3-NC groups was statistically significant (*P* < 0.01). To detect the Runx1t1 protein expression, total proteins were extracted in the three groups and subjected to semi-quantitative assays. The data showed that the protein level of Runx1t1 in the LV3-Runx1t1-RNAi group was obviously lowest of the three groups (Fig. 3b). These results illustrated that LV3-Runx1t1-RNAi markedly downregulated the Runx1t1 gene and protein expressions. To examine whether Runx1t1 knockdown decreased the neuronal differentiation of NSCs, cells were infected and then transferred to the renewed differentiation medium to evaluate neuronal differentiation. Ten days later, the differentiated cells were processed for Tuj1 staining. The mock group (Fig. 3c–e) and the LV3-NC group (Fig. 4f–h) revealed 11.32 ± 1.47% and 11.77 ± 3.01% Tuj1-positive cells, respectively. However, in the LV3-Runx1t1-RNAi group, less 5% cells (4.19 ± 0.95%) expressed Tuj1 (Fig. 3i–k) (*P* < 0.05; Fig. 3l).

Next, we also used LV4-Runx1t1 to induce Runx1t1 expression in vitro*.* In the overexpression experiments, the Runx1t1 gene in the Runx1t1 overexpression group was about 2.4-fold higher than that in the mock-infected group and 2.1-fold greater than that in the LV4-NC group (Fig. 4a). The level of Runx1t1 protein in the Runx1t1 overexpression group was also significantly elevated than that in the blank and LV4-NC groups (Fig. 4b). In order to further detect whether upregulation of Runx1t1 could promote the neuronal differentiation, NSCs were also transferred to the differentiation medium to evaluate neuronal differentiation. The results showed that in the Runx1t1 overexpression group, about 26% cells (26.26 ± 3.32%; Fig. 4i–l) was Tuj1-positive cells, which was significantly more than that in the blank (Fig. 4c–e, l) and LV4-NC groups (Fig. 4f–h, l).

### Distribution of Runx1t1 in rat hippocampal DG in vivo

**Fig. 5:**
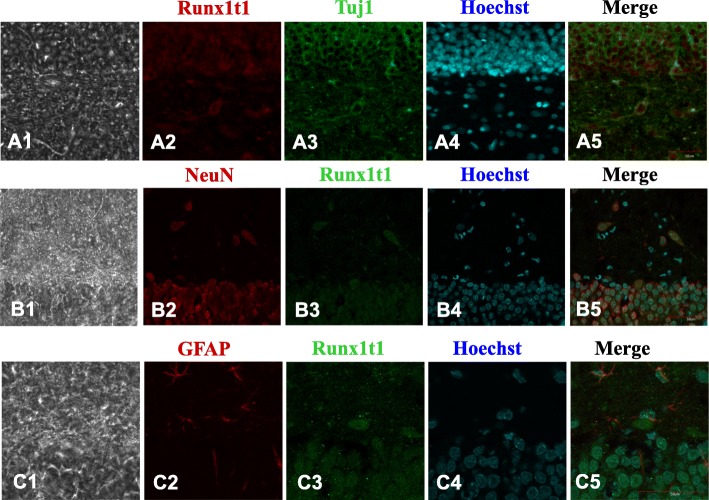
The neurons and astrocytes in rat hippocampal DG were labeled with Runx1t1. We found that Runx1t1 weakly expressed throughout the hippocampus (**A2**, **B3**, **C3**). The maximum number of Tuj1- and NeuN-positive cells expressed Runx1t1 in rat hippocampal DG (**A1**–**A5**, **B1**–**B5**). Contrastingly, no Runx1t1 expression was observed in the GFAP-positive cells (**C1**–**C5**). Scale bar = 50 μm

We also examined the Runx1t1 expression and distribution in rat hippocampal DG. The neurons and astrocytes were labeled with Runx1t1. Runx1t1 was found to be weakly expressed throughout the hippocampus (Fig. 5A2, B3, C3). A maximum number of the Tuj1- and NeuN-positive cells expressed Runx1t1 in rat hippocampal DG (Fig. 5A1–A5, B1–B5). However, Runx1t1 was not observed in the GFAP-positive cells (Fig. 5C1–C5). Thus, we concluded that Runx1t1 expression was localized in the neurons.

### Overexpression of Runx1t1 promotes the neurogenesis of hippocampus in vivo

**Fig. 6:**
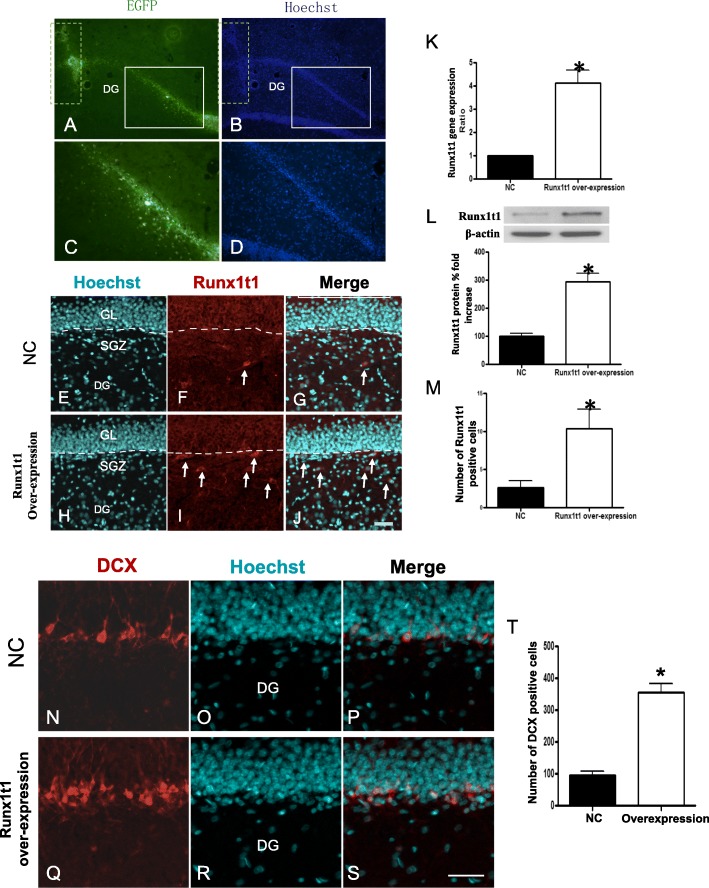
The dotted box showed the injection tract (**a**, **b**). Several EGFP-positive cells gathered near the track. The upper cells in the granular and subgranular zones of DG were transfected notably. Thus, the square region (shown in **a**–**d**) was the area of interest in immunofluorescence staining analyses for the subsequent studies. Real-time PCR revealed that Runx1t1 gene expression level in the LV4-Runx1t1 group was significantly higher than that in the LV4-NC group (**k**). The protein level of Runx1t1 in the LV4-Runx1t1 group was also higher than that in the LV4-NC group, which presented weak expression (**l**). The number of Runx1t1 immunopositive cells in the LV4-Runx1t1 group was also significantly than that in the LV4-NC groups (**e**–**j**, **m**). After 14 days, immunocytochemistry was performed. Extensive DCX immunoreactivity was detected in the LV4-Runx1t1 group (**n**–**s**). The number of DCX-positive cells was significantly more, and the cell processes were longer than that in the LV4-NC group (**t**). Scale bar = 50 μm, *P* < 0.05

To further demonstrate the role of Runx1t1 in neurogenesis of rat hippocampus in vivo, LV4-Runx1t1 lentivirus was injected into DG to upregulate Runx1t1 expression. Three days post-injection, coronal sections through hippocampus were directly observed under a fluorescence microscope. The dotted box showed the injection needle tract (Fig. 6a, b). Several EGFP-positive cells gathered around the track. The upper cells in the granular zone and SGZ of DG were transfected notably (Fig. 6a, b). The square region (Fig. 6a–d) was the object of research interest in immunofluorescence staining analyses for the subsequent studies. Real-time PCR demonstrated that the expression level of Runx1t1 gene in the LV4-Runx1t1 group was significantly higher than that in the LV4-NC group (Fig. 6k). The protein level of Runx1t1 in the LV4-Runx1t1 group was also greater than that in the LV4-NC group with weak expression (Fig. 6l). In addition, the number of Runx1t1-positive cells in the LV4-Runx1t1 group was significantly higher than that in the LV4-NC group (Fig. 6e–j, m).

Fourteen days later, immunohistochemistry was performed on the bilateral hippocampus using an anti-DCX antibody to observe the generation of newborn neurons in hippocampal DG. Extensive DCX immunoreactivity was detected in the LV4-Runx1t1 group (Fig. 6n–s); the number of DCX-positive cells was significantly increased, and the cell processes were longer and richer than that in the LV4-NC group (Fig. 6t). These results indicated that upregulating Runx1t1 expression could induce the neuronal differentiation in hippocampal DG.

## Discussion

The bHLH proteins promote neurogenesis by inducing changes in the gene expression required for neuronal differentiation [11, 12, 23], including the MTG genes. The MTG family is a small group of transcriptional repressors, which bridges various transcription factors severed as “protein scaffolds.” During neural development, members of the MTG family are involved in a negative feedback loop which regulates the normal progression of neurogenesis, when they are induced by bHLH transcription factors that reversely inhibit the activity of the bHLH proteins [24]. The MTG genes present the regional expression patterns in the developing nervous system. Aaker et al. [24] analyzed the differential expression patterns of MTG in the developing chick spinal cord, and they found that MTG could play a role in positively regulating neurogenesis in multiple cell types of the developing nervous system. Thus, in the developing chick spinal cord, inhibiting the function of MTG proteins reduced the number of cells which differentiate into neurons. Alternatively, the MTG gene expression may correspond to particular stages of neuronal maturation [24].

Runx1t1, also known as ETO or MTG8, is a transcription factor and a member of the MTG family. Runx1t1 was induced by NEUROG2 and repressed by ASCL1 [25], and its mRNA expression has been shown in several human tissues, with highest in the brain and heart [26]. The significant expression and the general clues provided by the protein sequence and structure suggested that Runx1t1 might act as a regulator to modulate development of the nervous system. Several studies have suggested that Runx1t1 was involved in the proliferation and affected the differentiation capacity of the hematopoietic progenitors [18, 27–29]. Despite extensive efforts to understand the function of Runx1t1 protein in the etiology of cancer, the lack of knowledge about their function in normal embryonic development continues to persist. In the adult brain, the ongoing neurogenesis convincingly occurred in the subventricular zone (SVZ) and SGZ of hippocampal DG. The hippocampal neurogenesis continuously generates new granular neurons; these newborn neurons integrate into the DG. In this study, the NSCs derived from the rat hippocampus and DG of the hippocampus were selected as candidates to investigate the correlation of Runx1t1 expression with neurogenesis in vitro and in vivo.

Studies on the expression patterns of Runx1t1 in developing chick and mouse nervous systems showed a dynamic ventral-to-dorsal shift according to the progress of development [24, 25]. Since neurogenesis occurs in a ventral-to-dorsal pattern, the dynamic shift in the pattern of Runx1t1 gene expression might be related to the pattern of neurogenesis [25]. Runx1t1 mRNA was also shown to be expressed in whole mouse embryos as early as embryo day 7 (E 7), peaked at day E 11, declined slightly, but continued to express at day E 17 [25]. The expression was abundant in the newborn mouse brain and started to decrease as the animal matured. In the current study, we observed that Runx1t1 was strongly expressed in NSCs cultured in vitro, begun to decline as the differentiation of NSCs occurred, and then maintained a weak expression in the neurons. During differentiation, Runx1t1 merely localized in the neuronal cells and was not expressed in astrocytes. In vivo, Runx1t1 expression was also assessed by immunofluorescence staining and was found to be weakly expressed throughout the hippocampal RG. Only the Tuj1- and NeuN-positive neurons expressed Runx1t1, whereas its expression was not observed in the GFAP-positive astrocytes. These results suggested that Runx1t1 might be associated with the neuronal differentiation.

Accordingly, the role of Runx1t1 expression in regulating the neuronal differentiation of NSCs was examined in vitro. After LV3-Runx1t1-RNAi efficiently knocked down Runx1t1 expression during the differentiation of hippocampal NSCs, we found that only 3.2% cells differentiated into Tuj1-positive neurons, less than that in the mock-infected cells and LV3-NC groups. Conversely, after LV4-Runx1t1 was used for the upregulation of Runx1t1 in the NSCs, > 30% cells differentiated into Tuj1-positive neurons. These results may indicate that low expression of Runx1t1 decreases neuronal differentiation of NSCs derived from the rat hippocampus, whereas high Runx1t1 expression promotes NSCs to differentiate into neurons in vitro. Therefore, we suggested that Runx1t1 was required by NSCs to undergo neuronal differentiation. In the previous studies, we also provided the experimental evidence that decreased Runx1t1 expression reduced the neuronal differentiation of the hippocampal radial glial cells (RGCs), one of the precursor cells of the hippocampus, and increased Runx1t1 expression caused a greater number of RGCs to differentiate into neurons [21]. In order to further demonstrate the role of Runx1t1 in neurogenesis of rat hippocampus in vivo, LV4-Runx1t1 lentivirus was injected into DG for the efficient upregulation of Runx1t1 expression. A large number of DCX-positive cells were detected in the LV4-Runx1t1 group; the cell processes were longer and richer than that in the LV4-NC group. These results indicated that upregulating Runx1t1 expression could induce the neuronal differentiation in hippocampal DG. Runx1t1 was speculated as a putative transcription factor containing two zinc-chelating domains. However, it was not able to bind specific DNA sequences and did not possess any DNA-binding ability. This phenomenon was mainly characterized by its four nervy homology regions (NHRs). These NHRs define the domains of Runx1t1 that mediate interactions with other proteins, such as the N-CoR/mSin3A/HDACs, to form a co-repressor complex for transcription repression [17, 30, 31]. During early adipogenesis, Runx1t1 may act as an inhibitor of C/EBPβ contributing to its characteristically delayed activation, thereby block transcription of its downstream genes (such as PPARγ), inhibiting preadipocyte differentiation [32]. Serving as a common angiogenic driver for vaculogenesis and functionality of endothelial lineage cells, Runx1t1 direct angiogenesis by activating a number of angiogenic factors (VEGFA, BMP4, and TGF-β2) [33]. Runx1t1 also is demonstrated to regulate pancreas development by regulating pancreatic polypeptide and ghrelin expression [34]. These findings reveal that, during the development and disease progression, Runx1t1 creates multiple effects on different cells. Therefore, we suggested that Runx1t1 could be involved in the process of neuronal differentiation during neurogenesis in the hippocampus by acting downstream of the proneural proteins as transcriptional factor.

## Conclusions

In summary, our findings have indicated that the expression of Runx1t1 in hippocampal NSCs decreased significantly after their differentiation, mainly localized in the neurons, and was not observed in the astrocytes in vitro and in vivo. Our results further proved that Runx1t1 plays a very important role in the process of hippocampal neurogenesis.

## Data Availability

The datasets used and/or analyzed during the current study are available from the corresponding author on reasonable request.

## Abbreviations

Runx1t1: Runt-related transcription factor 1 translocated to 1
NSCs: Neural stem cells
NPCs: Neural progenitor cells
DG: Dentate gyrus
MTGs: Myeloid translocation genes
bHLH: Basic helix-loop-helix
BrdU: 5-Bromo-2-deoxy-uridine
